# A Preliminary Single-Centre Study on the Risk Factors Associated with Persistent Feeding Disorders in Children

**DOI:** 10.3390/nu17071111

**Published:** 2025-03-22

**Authors:** Marta Ewelina Lis, Martyna Chojnacka, Ewa Łoś-Kiszkowiak, Beata Ziółkowska, Aneta Krogulska

**Affiliations:** 1 Student Research Club Paediatric, Allergology and Gastroenterology, Collegium Medicum in Bydgoszcz, Nicolaus Copernicus University in Toruń, 87-100 Toruń, Poland; 2 Department of Pediatrics, Allergology and Gastroenterology, Collegium Medicum in Bydgoszcz, Nicolaus Copernicus University, 87-100 Toruń, Poland; 3 Faculty of Psychology, Kazimierz Wielki University, 85-064 Bydgoszcz, Poland

**Keywords:** paediatric feeding disorders, child nutrition, feeding difficulties

## Abstract

**Background/Objectives**: The epidemiology of childhood feeding disorders (PFDs) reveals a significant prevalence. The aim of the study was to identify risk factors for the persistence of PFDs. The study considered psychosocial and biological factors. **Methods**: A retrospective cross-sectional study was performed at two time points (Stage I and II); the mean interval was three years. The study included 56 children hospitalised between 2013 and 2023. Participants were divided into Group A (*n* = 39) and Group B (*n* = 17). Group A included children whose feeding disorders persisted until Stage II, while Group B included children whose symptoms of feeding disorders were no longer noted at Stage II. The mean age of children in Stage I was 4.5 ± 4.3 years in Group A and 6.25 ± 6.1 years in Group B. In Stage II, the mean age was 7.76 ± 5.3 years in Group A and 9.4 ± 6.7 years in Group B. **Results**: In Stage I (Groups A + B), 22 (39.29%) children refused to eat all foods, 26 (46.43%) consumed fewer foods than in the previous period, 19 (33.93%) ate only at night, and 12 (21.43%) consumed only selected food. A significant difference in the prevalence of wasting was noted at Stage II (Group A *n* = 19, 48.7% vs. Group B *n* = 3, 17.6%, *p* = 0.029). Feeding difficulties were found to start during exclusive breastfeeding in 28.6% in Group B but only in 10.8% in Group A. Feeding disorders concerning foods other than milk were significantly more common in Group A (*n* = 31; 83.8%) than Group B (*n* = 6; 42.9%; *p* = 0.011). At the end of one year of age, foods of all consistencies were consumed more often by children in Group B (*n* = 9; 64.3% vs. *n* = 10; 27%; *p* = 0.036). **Conclusions**: Children with feeding disorders comprise a heterogeneous group of patients. Those who only present feeding disorders associated with the consumption of milk and who consume foods of different consistencies by the end of one year of age demonstrate a better prognosis.

## 1. Introduction

Food intake is a complex process requiring the interaction of the central and peripheral nervous systems, the cardiorespiratory system, the oropharyngeal mechanism, and the gastrointestinal tract, with the support of the craniofacial and musculoskeletal structures. For children, the functioning of the parent or carer as a feeder plays a key role. Disruption of any of these biological or relational systems increases the risk of developing feeding disorders and related complications [1]. Disorders affecting biological systems include, among others, anatomical defects in the oral, nasal, or pharyngeal cavity, such as cleft palate, cleft lip, and dental diseases [1,2]. The development of feeding disorders may also be related to numerous defects and diseases of the gastrointestinal tract and respiratory tract, such as allergies and food intolerances, gastrointestinal motility disorders, vocal cord or fold paralysis, and congenital anomalies of both systems [1,2,3,4,5]. Disorders of the nervous system that play a significant role in the development of feeding disorders are primarily motor control disorders with reduced or increased muscle tone, as well as autism spectrum disorders. The influence of iatrogenic causes, such as long and/or repeated hospitalisations (especially those related to intensive care), as well as invasive medical procedures, such as gastrointestinal surgery, should not be overlooked. Conditions that are not usually immediately associated with an increased risk of feeding disorders and the development of their complications are cardiopulmonary disorders. These include conditions such as heart failure of various causes and bronchopulmonary dysplasia [1,2,4]. In some children, the disorders do not occur alone, which may lead particularly to the development and maintenance of eating disorders. The caregiver–child relationship associated with feeding is an important part of the proper feeding process. Its disruption is one of the possible psychosocial dysfunctions mentioned in diagnostic criteria for PFD [1,2,3,4,5].

Therefore, feeding disorders require a multidisciplinary approach to their treatment. However, while 25% to 45% of parents report feeding problems with their children [1,6], only 1 to 5% are diagnosed with feeding disorders [2,7,8]. It is important to distinguish between feeding disorders and feeding difficulties. While the former denotes a serious problem in food intake that causes significant physiological, nutritional, or emotional consequences in the child, the latter is a generic term indicating the existence of some form of problem with eating (“if the mother says there is a problem, that is, there is a problem”) [2].

Feeding disorders have been associated with other conditions. For instance, they have been diagnosed in 30% of babies born prematurely, about 80% of patients with neurological diseases or malformations [3,4,5,9,10], and 30 to 40% of children with cerebral palsy [11]. They have also been identified in up to 90% of autistic children [2,12], and it has been estimated that 40–70% of children with chronic medical concerns (e.g., congenital or acquired respiratory, cardiac, and gastrointestinal problems and developmental disabilities) experience feeding difficulties [13,14].

In 2019, paediatric feeding disorder (PFD) was formally defined as an impairment of oral intake that is not age appropriate and is associated with one or more dysfunctional areas among medical, nutritional, feeding skills, and psychosocial domains. In this sense, functional impairment is understood based on the definition given by the World Health Organization (WHO) International Classification of Functioning, Disability and Health (ICF) [15]. However, PFDs also include significant disturbances in nutrient and caloric intake beyond normal fluctuations in hunger, food preference, and/or interest in eating [11]. A diagnosis of a PFD requires symptoms to occur daily for at least two weeks, with acute PFD characterised by a course shorter than three months, and chronic PFD by one longer than three months [15].

Regarding the domains of influence of PFDs, the medical domain includes anatomical, neurological, cardiorespiratory, digestive, and developmental problems. The nutritional domain mainly concerns the quantity, quality, and variety of food consumed, which may result in malnutrition or micronutrient deficiency; approximately 25–50% of children with PFD experience malnutrition, particularly chronically ill patients and those with neurodevelopmental disorders [9,10]. The feeding skills domain concerns neurodevelopmental delays; these can manifest at any time during the first years of life and are related to changes in oral and pharyngeal anatomy, neuromuscular coordination, resultant neurological deficits or adverse feeding experiences [16,17,18]. Finally, the psychosocial domain concerns the interaction between the child and their environment; dysfunctions are believed to result from a mismatch between the child’s capabilities and the caregiver’s expectations, health problems of a family member, or environmental factors such as a distracting feeding environment and inconsistent meal schedules.

While PFD is a relatively common clinical diagnosis, its prevalence is increasing [19]. However, generally speaking, no universally accepted classification currently exists, and the condition is used to refer to a heterogeneous group of conditions including poor appetite, food selectivity, food refusal, and delayed or absent development of feeding skills, which may or may not be accompanied by inappropriate growth [20]. Some cases are easily identified, while others are more challenging, particularly in the presence of normal growth. The most common symptoms include malnutrition, refusal to eat and drink, food pocketing, disruptive feeding behaviour, slow feeding (>30 min to finish), food selectivity or rigid food preferences, limited appetite, and delayed feeding milestones [1].

It should be emphasised that the diagnosis of PFD is difficult and that PFDs can sometimes be misdiagnosed as ARFID (Avoidant/Restrictive Food Intake Disorder); however, the two can be differentiated by the fact that ARFID includes behavioural elements, while PFD adds medical, nutritional, and skills data [15]. Noel suggests that for the sake of simplicity, ARFID and PFD can be subsumed together as ARFID/PFD and used to simply refer to the feeding disorder that is central to both concepts [21]. Patients with ARFID/PFD commonly experience gastrointestinal dysfunction and anxiety. It is worth noting that medical conditions can mask eating disorders, and vice versa [21].

Although publications by Polish authors on PFDs are available [22,23,24,25,26,27,28,29,30,31], both its growing incidence among children and the need to broaden our own research and practical competences drove the implementation of the present study. Furthermore, no studies to date have performed any long-term evaluation of children with feeding disorders. Some researchers are of the opinion that eating disorders are transitional problems [30,31], while others believe them to be chronic conditions that tend to worsen over time [1,32].

Hence, the aim of the study was to identify risk factors for the persistence of feeding disorders in children. The study considered both psychosocial factors, especially family factors, and biological factors that could be related to the occurrence of feeding disorders in children.

## 2. Materials and Methods

The study received permission from the CM NCU Ethics Committee in Bydgoszcz, Poland (Nr 451/2022). The present research consisted of a retrospective–prospective, cross-sectional study performed at two time points: at the moment of first hospitalisation (Stage I) and at a later survey (Stage II), i.e., in the period 1 June 2022 to 2 February 2023; the mean interval between Stage I and II was three years. The study included 56 children hospitalised at the Department of Paediatrics, Allergology and Gastroenterology, University Hospital No. 1 in Bydgoszcz, Poland (the regional tertiary referral center for the assessment of feeding difficulties) between 2013 and 2023. The mean age of children in Stage I was 4.5 ± 4.3 years in Group A and 6.25 ± 6.1 years in Group B. In Stage II, the mean age was 7.76 ± 5.3 years in Group A and 9.4 ± 6.7 years in Group B.

Although PFD has been recently defined, no specific single International Classification of Diseases (ICD) code is available to capture all children with feeding disorders [15]. As such, the present study uses a variety of ICD codes to capture children with a diagnosis of PFD. Feeding difficulties included feeding disorders (F98.2; F50.8, F50.9, R63.3, F50.89/ICD-10-CM).

A feeding disorder team including a paediatrician, gastroenterologist, psychologist, dietician, neurologist, and rehabilitation therapist was established at the clinic in 2019. Since then, the diagnosis of PFD was based on the accepted diagnostic criteria. PFD was diagnosed according to the consensus definition as a disturbance in the age-appropriate oral intake of nutrients, lasting for at least two weeks, an absence of cognitive processes consistent with eating disorders, and a disturbed pattern of oral intake not due to a lack of food or incongruence with cultural norms [4].

The participants were divided into two groups: Group A (39 children), consisting of children whose feeding disorders persisted from Stage I of the study and continued into Stage II, and Group B (17 children) whose feeding disorders were present in Stage I but resolved by Stage II.

The study was based on the analysis of the children’s data from hospital records (Stage I) and a self-administered survey questionnaire sent to the parents by email (Stage II). The survey questionnaire contained 86 questions: 56 closed and 30 open. The obtained data were used to assess environmental factors potentially related to the development of feeding disorders and to compare data from Stages I and II. The whole assessment included patient parameters from the hospitalisation period, the perinatal data, demographic and socio-economic data of the children, as well as their nutrition, medical history, and information about first-degree relatives.

In addition, physical development parameters were assessed. The child’s weight, height, and body mass index (BMI) were converted into standard deviation scores (z-scores) based on the WHO centiles and z-scores for height-for-age, weight-for-age, and BMI-for-age [33]. The data were interpreted based on WHO Global Database on Child Growth and Malnutrition recommendations [34,35]. Underweight was defined as weight-for-age < −2 standard deviations (SD) of the WHO Child Growth Standards median, stunted growth as height-for-age < −2 SD of the WHO standard, and wasting as BMI < −2 SD of the WHO standard [35].

**Figure 1:**
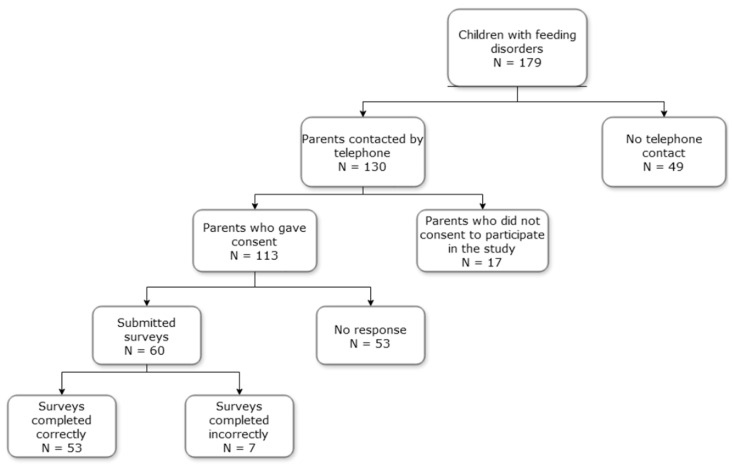
A flow chart of the survey.

Consent for the survey was obtained during a telephone call prior to the survey being conducted. Of the 179 people approached for participation in the study, contact was made with 130. Of these, 113 people consented to take part, and were sent the survey questionnaire. Responses were received from 60 people, of which 53 were correctly completed and included in the analyses. Three of the seven incorrectly completed surveys qualified for the study because the only missing data in these surveys was information about the parents in the case of adopted children. The remaining 4 surveys contained too much incomplete information and were therefore rejected. A flow chart of the survey is shown in Figure 1.

Statistical analysis: The analysis was performed using Python 3.8 with the following libraries: matplotlib (v. 3.7.1), numpy (v. 1.24.3), pandas (v. 2.0.2), pingouin (v. 0.5.3), seaborn (v. 0.12.2), and statsmodels (v. 0.14.0). In addition, PS IMAGO PRO 10 along with the IBM SPSS Statistics 29 engine was used. A *p*-value below 0.05 was assumed to indicate statistical significance. Nominal and ordinal variables are presented in tables as Variable, *n* (%). Continuous variables are described as Variable, mean ± SD.

The following statistical tests were used:

1. To check whether the distribution of the variable comes from the normal distribution (assumption required to conduct the Student’s *t*-test), the Shapiro–Wilk test was used.
2. To compare continuous variables in two groups, the following were used:
  - Student’s *t*-test (with Welch’s correction) for two independent samples, when the assumption of normal distribution was met in the groups or the sample size was ≥30;
  - Two-sided nonparametric Mann–Whitney U test, otherwise.
3. To compare nominal variables in two groups, the following were used:
  - Asymptotic chi-square test (with Yates’s correction) in a situation where the expected value of cells are 5 or greater;
  - Fisher’s exact test, when the assumptions of the asymptotic chi-square test are not met.
4. To compare ordinal variables in two groups, the Cochran–Armitage test for trend was used.

## 3. Results

First, the analysis confirmed whether feeding disorders were present in the studied children based on parental accounts and documentation. It was found that in Groups A + B (*n* = 56), during Stage I, 22 (39.29%) children refused to eat all foods, 26 (46.43%) children consumed significantly fewer foods compared to the previous period and/or peers, 19 (33.93%) ate only at night, and 12 (21.43%) consumed only selected food. No significant differences in the prevalence of feeding distractions were observed between Groups A and B. In 14 (25%) children from Groups A and B, the parents reported using distractors (TV, phone) during feeding. Fourteen children (25%) required nasogastric tube feeding, and 12 (21.43%) demonstrated whooping during feeding.

The characteristics of Groups A and B at Stages I and II are given in Table 1, together with data on their physical development. No statistically significant differences were found between Groups A and B for either stage; however, a significant difference in the prevalence of wasting was noted at Stage II, with 19 cases noted in Group A (48.7%) compared to three in Group B (17.6%) (*p* = 0.029).

The perinatal data is given in Table 2. The mean birth weight in Group A was only slightly lower than that of Group B (*p* = 0.176). Most children were born by natural childbirth, i.e., 22 (56.4%) in Group A and nine (52.9%) in Group B (*p* = 0.958). Abnormal pregnancies were recorded in approximately half of the children in Group A (*n* = 21, 53.8%) and one-third in Group B (*n* = 5, 29.4%) (*p* = 0.163). No significant differences were found regarding the duration and sequence of pregnancy, postpartum status, or exposure to nicotine smoke.

The majority of children in both Groups A and B were from urban areas; however, the children in Group A enjoyed significantly more dwelling space per person than Group B (21.3 ± 11.47 vs. 16.5 ± 10.22; *p* = 0.026). In two cases from Group A (5.1%) and one from Group B (5.9%) (*p* = 0.972), the parents described their own material situation as difficult. Most of the children lived with both parents, including 29 (74.4%) in Group A and 14 (82.4%) in Group B (*p* = 1). Most children had siblings, including 24 (61.5%) in Group A and 12 (70.6%) in Group B. No significant differences were observed in the number or type of carers (mother, father) living with the child, the main source of income of the mother and father, or the age of the siblings (Table 3).

Most of the mothers and fathers in both groups had a university education (mothers: 17; 47.2% vs. 9; 52.9%, *p* = 0.607; fathers: 15; 41.7% vs. 7; 41.2%, *p* = 0.761). No significant differences in age and physical development were found between the groups for either parent (Supplementary Table S1).

No significant intergroup differences in the prevalence of early childhood feeding disorders (’non-eater’) or selective appetite were noted in the parents, although a higher prevalence of picky eaters was found among mothers in Group A compared to those in Group B (*n* = 17; 43.6% vs. *n* = 5; 29.4% mothers, respectively) (*p* = 0.483) (Table 4).

The feeding data during the first year of life were also assessed. During the first three days of life, 11 children in Group B (64.7%) were fed exclusively with breast milk, compared to 18 children in Group A (46.2%) (*p* = 0.372). Interestingly, while more than twice the proportion (*n* = 10; 25.6%) of babies in Group A were fed exclusively with artificial mixtures during the first three days of life than in Group B (*n* = 2; 11.8%), the difference was not statistically significant (*p* = 0.37). From the fourth day of life onwards, most children in both groups were mix-fed (*n* = 16; 41% and n = 8; 47.1%) (*p* = 0.758). Thirteen children in Group A, i.e., 33.3%, were fed a milk-substitute mixture, compared to only one (5.9%) child in Group B (*p* = 0.109).

Approximately one-third of the children in both groups were exclusively fed naturally up to six months of age (*n* = 12; 30.8% and *n* = 6; 35.3%). The mean duration of breastfeeding in Group A was slightly longer (13.18 ± 13.08 months) than in Group B (11.57 ± 9.98), although this difference was not significant (*p* = 1). No differences in the prevalence of lactation problems were reported between the groups.

The mothers in Group A introduced complementary foods later than those in Group B, but non-significantly so (7.59 ± 7.34 and 6.36 ± 1.69 months, respectively) (*p* = 0.748). At the end of one year of age, foods of all possible consistencies were consumed significantly more often by children in Group B (*n* = 9; 64.3%) than Group A (*n* = 10; 27%) (*p* = 0.036), and exclusive consumption of liquid food was only noted in seven (18.9%) children in Group A compared to none in Group B.

Feeding disorders regarding milk consumption were almost equally common in the two groups (*n* = 16; 43.2% for Group A and *n* = 6; 42.9% for Group B) (*p* = 0.770). Feeding disorders concerning foods other than milk were significantly more common in Group A (*n* = 31; 83.8%) than Group B (*n* = 6; 42.9%) (*p* = 0.011). At the end of one year of life, 12 children in Group A (32.4%) were only consuming dairy products, compared to only one child (7.1%) in Group B (*p* = 0.184). In addition, 16 (43.2%) children in Group A were consuming exclusively selected foods compared to eight (57.1%) in Group B (*p* = 0.184).

Feeding difficulties were found to start during exclusive breastfeeding in almost one-third of the children (*n* = 4; 28.6%) in Group B but only 10.8% (also *n* = 4) in Group A and during only complementary foods in four (28.6%) children in Group B and nine (24.3%) in Group A (*p* = 0.374). Feeding problems began when feeding modified milk and complementary foods in more than one-third of the children in Group A (*n* = 13; 35.1%) compared to 21.4% in Group B (*n* = 3). No significant differences were found between Group A and Group B in terms of whooping during feeds, excessive urination, recurrent vomiting, mean feeding time, dummy sucking, and the use of teethers. Feeding disorders were preceded by an upper respiratory or urinary tract infection in only four (10.8%) children in Group A and none in Group B. Feeding by nasogastric tube or G-tubes during lifetime was reported in 11 (29.7%) children from Group A and three (21.4%) from Group B (*p* = 0.73) (Supplementary Table S2).

Data on the comorbidities in the examined children, and the specialist support provided to patients and their families, are described in Table 5. The presence of comorbidities was demonstrated in 34 (60.7%) of the examined children, including 26 (66.7%) in Group A and 8 (47%) in Group B (*p* = 0.278). No significant differences with regard to the type of comorbidities or the frequency or type of specialised assistance received by the child and the parents were noted. According to both the mothers and fathers, their relationship with the child was satisfactory.

## 4. Discussion

Feeding disorders are an important problem during child development, and one that affects both children and their families, but they are relatively poorly understood in Poland.

Our findings indicate that among children at one year of age, a better prognosis, i.e., resolution of feeding disorders, was obtained when feeding disorders were associated only with milk consumption, or when foods of different consistencies were consumed. However, it was not possible to identify other personal and environmental factors that increase the risk of the occurrence and persistence of feeding disorders in children.

PFD is most prevalent in children under the age of 5 years, but continues to be common throughout childhood [19]. It is believed that PFDs and ARFID affect children of all ages [21]. Similarly, in the present study, the age of children at the time of diagnosis of feeding disorders varied, ranging from 0.2 to 16 years of age. Upon the analysis of 415 studies, Estrem et al. indicated that 318 studies (77%) reported the mean value and the range of the age of their sample (mean = 4.4 years, SD = 3.7, range = 0–17 years) [36]. Galai et al. demonstrated that age at presentation PFD emerges as a significant factor in determining the patterns of the PFD. Treatment planning should be based upon the patient’s age. In the younger age group, the focus should be upon the development of feeding skills. However, in the older age group, the focus should be on nutritional assessment and recommendations by a specialist dietician [37].

Data on the influence of sex on feeding disorders are inconclusive. For example, Kovacic et al. and Galai et al. indicate that for unclear reasons, PFD is more frequent in boys [19,37]. In contrast, Cooney et al. and Cucinotta et al. report the majority of children with ARFID were girls [38,39]. In the present study, based on patients with PFDs, girls predominated.

Based on our own results, there were no group differences in perinatal factors like gestational age, method of birth, course of pregnancy, and birth weight. The similar results were shown by Galai et al. [20]; however, the same authors two years earlier showed that low birth weight was significantly more frequent in children with PFDs [40].

Our findings indicate that children with persistent feeding disorders (Group A) were more likely to experience problems with the intake of foods other than milk, i.e., complementary foods, at one year of age (*p* = 0.01). According to the caregivers, as many as 83.8% of children in this group refused to consume a significant part, or all, of the complementary foods in an appropriate quantity, and 43.2% demonstrated feeding disorders related to milk consumption. The fact that a similar proportion of children in Group B (42.9%) also exhibited problems with milk may suggest that this issue, without any co-occurrence of problems with intake of other foods, is not a significant predictor of the persistence of later feeding disorders.

The texture and consistency of food have a significant influence on its attractiveness to children. Our findings indicate that among children of one year of age, those with persistent feeding disorders were more likely to prefer a liquid or pulpy consistency compared to other children (*p* = 0.04). The reluctance to eat foods with a consistency other than liquid or mushy can be explained by sensory integration disorder (SID). It is possible that SID may also result in some children’s preference for only dairy foods or a selective intake of only odour-neutral foods. Other possible reasons for the reluctance to eat harder foods include anatomical defects, e.g., in the craniofacial region, or malocclusion in older children. It is, however, worth pointing out that malocclusion sometimes arises due to dietary mistakes in early childhood, such as delaying the introduction of foods with different textures, or containing lumps. It may be that the consumption of foods with a variety of textures and consistencies, even by children demonstrating some food selectivity or consuming insufficient food, is a positive predictor of that feeding disorders may recede, as reflected in our own study.

It is worth bearing in mind that food selectivity and food restriction can be associated with food neophobia, which should be distinguished from the child’s refusal to eat in order to assert autonomy [41]. Indeed, it is suggested that food neophobia is not a common phenomenon in children aged 1–2 years but becomes more pronounced later in childhood [41,42].

Feeding disorders in children are multifactorial (e.g., birth weight, age, sensory sensitivity) and therefore need to be considered holistically [42].

Our findings suggest that problems with the intake of complementary foods in infants are indicative of a higher risk of feeding disorders persisting with age, irrespective of a previous or co-occurring problem with milk consumption.

They also indicate that in children whose feeding disorders had resolved (Group B), almost three times as many children presented feeding disorders exclusively during natural feeding. On average, mothers of children in this group breastfed for slightly less time than mothers of children with persistent feeding disorders (Group A). Moreover, the mothers in Group B were slightly more likely to have lactation problems. It is possible that the shorter breastfeeding duration was a direct result of the feeding disorder, but this is not certain. The most common lactation problem reported in a primary care office is the concern about a shortage of breast milk and/or its nutritional value, although this is again unclear [43]. Therefore, it is difficult to make a clear assessment of the scale of the problem on the basis of the survey results. Galai et al. showed that the absence of breastfeeding was more frequent in children with PFDs [40]; however, a study two years later did not confirm this observation [37]. Meanwhile, an analysis of 21 observational studies found the length and manner of breastfeeding or age of the child at the time of the introduction of complementary foods do not appear to have any significant impact on the occurrence of feeding difficulties at over one year of age [23].

Although breastfeeding is the best and the most natural form of nutrition, it is not without difficulties, so it often needs support [44,45,46]. Studies show that breastfeeding can be a challenge for the mother and, as a result, cause the child to have difficulty eating, because PFD results, among other things, from the child’s inability to feed [46]. Meanwhile, Bugaeva et al. claim that breastfeeding protects children from various mental disorders (including eating disorders) [47]. At the same time, breastfeeding can strengthen the mother–child attachment bond, and thanks to a secure attachment relationship, the mother is more calm and attentive and responds adequately to the child’s needs [48]. It is worth emphasising that difficulty breastfeeding does not mean a child will develop a PFD. Studies have shown that breastfeeding duration is positively associated with later food variety and healthy eating habits in children under five years [49,50].

Previous research has been shown that children with PFDs are significantly more likely to be of low socioeconomic status [40]; however, this has not been confirmed in other studies [20]. Our present findings do not indicate any significant differences in sociodemographic indicators between Groups A and B; we only noted that children with persistent eating disorders lived with their parents in larger dwellings. Interestingly, a study from Denmark found that higher parental socioeconomic status was associated with increased risk of eating disorders in offspring, particularly anorexia nervosa [51]. It is likely, however, that the parenting style of the carers may play a greater role than socio-economic status. Our own data do not suggest an association between the number of people living with the child, age, physical development parameters, or the presence of eating disorders in childhood in parents and the persistence of feeding disorders in children.

It has been proposed that parental childhood feeding history may provide important clues to PFD in the child [23,37,52]. There is only one published study on the association between a parental history of PFD and their children’s feeding disorders [37]. In the present study, 34 of the parents (60.7%) also report experiencing feeding difficulties during childhood. Galai et al. showed that 42.5% of children had parents who reported having PFD [37]. This apparent persistence of PFD between generations may be attributed to genetic factors and to the fact that the parents’ eating behaviour experiences may shape their children’s lifelong eating habits [53].

In our study, most parents of children with PFDs had an academic education. According to Galai et al., academic achievement and perfectionism of parents are associated with their children’s eating disorders [37]. Brytek-Matera et al. showed that both the maternal feeding style and core behavioural features of eating disorders were associated with avoidant/restrictive food intake disorder symptoms among 2- to 10-year-old children [52].

Most children with feeding disorders demonstrated coexisting food allergies, epilepsy, autism, oral and pharyngeal anatomical abnormalities, or CP. In children on the autism spectrum, SID is probably the most common cause of food avoidance; however, this does not exclude a genetic component that may directly contribute to the onset of feeding disorders in this group. Other studies have also confirmed that PFD is significantly more common in the population of children with coexisting malformations or chronic diseases [14,54].

In the present study, 25% of children were fed by nasogastric tube/G-tubes. The vast majority of children with G-tubes do have a feeding problem [15]. More than one-quarter of the babies in the present study (26.8%) were born prematurely. Brosig et al. report that 35% of children with ARFID were prematurely born [55].

Feeding disorders entail a number of consequences and affect both the child, due to impaired growth, nutritional deficiencies, dependence on enteral feeding, or impaired social functioning, and their friends and family. Restriction of food intake is often associated with high levels of stress for the carer, as well as comorbid psychiatric illnesses, social difficulties for the children, and impaired functioning of the family as a unit [14]. Based on the present data, both patients and their family members were more likely to have received specialist psychological, psychiatric, or dietary support.

As noted in a review by Cucinotta et al. [39], our present study confirmed the presence of wasting in more than half of the children at the time of diagnosis. Given that inpatient therapy is indicated in patients with weight deficiency [2], this observation is not unexpected; likewise, children whose feeding disorders had resolved at Stage II of the study were found to be more likely to have higher BMI values due to increased food intake.

A strength of the study is that it obtained data on feeding disorders in children from the Kujawsko-Pomorskie voivodeship at two time points. It also identified risk factors for the development and persistence of feeding disorders in children. So far, none of the follow-up studies performed in children with feeding disorders have been performed over a period longer than six months [36,39]. In addition, the presented study includes a simultaneous analysis of the presence of feeding disorders in the parents, an analysis that has only been conducted in one paper to date [37].

The study identified only three factors that were significantly associated with the persistence of feeding disorders: the type of food consumed at the end of one year of life, its consistency, and the area of the residence. However, this may be due to the small size of the study group and the retrospective nature of the study, although a review of 415 articles on PFDs found that 47.6% of the studies had a sample size smaller than 50 people [36].

Unfortunately, this study has some limitations. The data were acquired from a single centre, the group of children participating in the study is quite small, the results show few significant differences between stages in both groups, the feeding disorder team was not included from the beginning of the study, and the data were collected using a self-administered questionnaire. Moreover, similar to Galai et al., the parental PFD diagnosis was made based upon self-reporting, which could lead to associated recall bias or inaccuracy due to parental memory. Similarly to Galai et al. [37], some degree of incompatibility between clinical diagnosis and the formal PFD new definition may be also anticipated. However, since the diagnosis of PFD was made by a multidisciplinary team comprised of a paediatric gastroenterologist, dietician, speech therapist, and psychologist, we believe that inaccuracies in the diagnosis of PFD were held to a minimum. Lastly, the patient cohort accessed care at a subspecialty clinic, and they may not represent the broader paediatric population. However, our hospital is a tertiary care medical centre, which we believe represents the general paediatric population. In addition, the study did not verify how parental care was provided; however, this will be checked in the next phase of the project.

Unrecognised PFDs can result in severe consequences, including a compromised immune system, chronic aspiration, growth failure, and death. Therefore, early identification and intervention are critical [15]. There is still no ideal tool that could be used to detect and monitor patients with this disease entity, and no data have been identified that would allow the creation of management algorithms. Effective prevention and treatment requires the education of the medical community as well as those in the immediate environment of children affected by feeding disorders. As such, successful and satisfactory treatment may be best provided through a multidisciplinary approach comprising medical, dietetic, speech therapy, psychological, and physiotherapeutic support. Further longitudinal studies are needed in order to better assess the outcome of specific therapeutic modalities [37]. Studies that report the long-term effects of intervention are also needed.

## 5. Conclusions

Children with feeding disorders comprise a heterogeneous group of patients. Those who only present feeding disorders associated with the consumption of milk and those who consume foods of different consistencies demonstrate a better prognosis, i.e., a faster resolution of feeding disorders, by the end of one year of age. The problem of feeding disorders requires further long-term research.

## Figures and Tables

**Table 1:** The characteristics of Group A and Group B at Stage I and Stage II.

Parameter	Group A *n* = 39 (100%)		Group B *n* = 17 (100%)		*p*	
Sex of the child, *n* (%) - Boy - Girl	17 (43.6) 22 (56.4)		6 (35.3) 11 (64.7)		0.776	
Prematurity, *n* (%)	9 (23.1)		6 (35.3)		0.349	
Parameter	Stage I	Stage II	Stage I	Stage II	*p* *	*p* **
Age, years Mean ± SD Min–max	4.5 ± 4.3 0.2–14	7.76 ± 5.3 0.8–17	6.25 ± 6.1 0.2–16	9.4 ± 6.7 1.5–19	0.851	0.375
Mean body mass, z-score ± SD Min–max	−1.93 ± 1.18 −4.47–0.75	−1.53 ± 1.23 −3.42–0.97	−2.33 ± 1.47 −5.74–−0.07	−1.12 ± 1.28 −3.04–0.24	0.440	0.423
Mean height, z-score ± SD Min–max	−0.75 ± 1.66 −5.12–3.89	−0.75 ± 1.97 −6.15–3.15	−0.92 ± 2.07 −5.26–2.71	−0.52 ± 1.63 −3.52–2.58	0.779	0.643
Mean BMI ^1^, z-score ± SD Min–max	−2.16 ± 1.45 −7.2–1.17	−1.97 ± 1.79 −5.42–2.65	−2.93 ± 1.53 −5.56–−0.64	−1.43 ± 1.87 −6.04–1.35	0.089	0.322
Underweight, *n* (%)	13 (39.4%)	10 (41.7%)	7 (70%)	3 (33.3%)	0.148	1.000
Stunted growth, *n* (%)	7 (17.9%)	9 (23.1%)	4 (23.5%)	3 (17.6%)	0.719	0.738
Wasting, *n* (%)	18 (46.2%)	19 (48.7%)	11 (64.7%)	3 (17.6%)	0.201	0.029

^1^ BMI—Body mass index. * Stage I-Group A vs. Group B; ** Stage II -Group A vs. Group B. Due to the inability to determine the Z-score values for the weight of some patients, the comparison of mean values for this variable was conducted on a reduced dataset. A total of 33 and 24 patients from Group A were included in Stages I and II, respectively, as well as 10 and 9 patients from Group B in Stages I and II, respectively.

**Table 2:** Perinatal data depending on the persistence of feeding disorders.

**Parameter**	**Group A** ***n* = 39 (100%)**	**Group B** ***n* = 17 (100%)**	***p***
Birth weight, grams			0.176
Mean ± SD	2655.90 ± 840.36	2960.59 ± 657.27	
Min–max	750.0–3950.0	1440.0–3700.0	
Hbd. grav. ^1^			0.578
Mean ± SD	37.23 ± 4.35	38.35 ± 2.74	
Min–max	25.0–41.0	32.0–42.0	
Type of birth, *n* (%)			0.958
- Vaginal delivery	22 (56.4)	9 (52.9)	
- Caesarean delivery	17 (43.6)	8 (47.1)	
Pregnancy number, *n* (%)			0.743
- First	18 (46.2)	8 (47.1)	
- Second	9 (23.1)	5 (29.4)	
- ≥Third	12 (10.3)	4 (23.5)	
Birth order, *n* (%)			0.844
- First	21 (53.8)	8 (47.1)	
- Second	9 (23.1)	7 (41.2)	
- ≥Third	9 (23.1)	2 (11.8)	
Apgar score, *n* (%)			0.703
- ≤4	5 (12.8)	1 (5.9)	
- 5–7	2 (5.1)	2 (11.8)	
- 8–10	32 (82.1)	14 (64.7)	
Course of pregnancy, *n* (%)			0.163
- Abnormal	21 (53.8)	5 (29.4)	
- Normal	18 (46.2)	12 (70.6)	
Cause of abnormal pregnancy course, *n* (%)			
- Hypothyroidism	2 (5.1)	0 (0)	
- Hashimoto’s thyroiditis	2 (5.1)	0 (0)	
- IUGR ^2^	6 (15.4)	1 (5.9)	
- Anaemia	4 (10.3)	0 (0)	
- Polyhydramnios	2 (5.1)	2 (11.8)	
- Oligohydramnios	2 (5.1)	0 (0)	
- Diabetes	2 (5.1)	1 (5.9)	
- Infection	2 (5.1)	0 (0)	
- Single or multiple congenital defects	4 (10.3)	1 (5.9)	
- Other	8 (20.5)	3 (17.6)	
Exposure to tobacco smoke during pregnancy, *n* (%)			0.738
- Yes	9 (23.1)	3 (17.6)	
- No	30 (76.9)	14 (82.4)	
Form of exposure, *n* (%)			1.000
- Active smoking	3 (7.7)	1 (5.9)	
- Passive smoking	6 (15.4)	2 (11.8)	

^1^ Hbd. grav.—Hebdomas graviditatis (week of pregnancy); ^2^ IUGR—Intrauterine growth restriction.

**Table 3:** Demographic and socio-economic data depending on the persistence of feeding disorders.

Parameter	Group A *n* = 39 (100%)	Group B *n* = 17 (100%)	*p*
Place of residence, *n* (%)			0.261
- Village	17 (43.6)	6 (35.3)	
- Town with up to 50,000 inhabitants	6 (15.4)	1 (5.9)	
- Town with 50,000 to 100,000 inhabitants	8 (20.5)	2 (11.8)	
- Town with 100,000 to 500,000 inhabitants	6 (15.4)	4 (23.5)	
- City with over 500,000 inhabitants	2 (5.1)	4 (23.5)	
Area of the house/apartment where the child resides, per person, m^2^			0.026
Mean ± SD	21.30 ± 11.47	16.5 ± 10.22	
Min–max	8.29–83.33	6.75–46.67	
Number of people living with the child			0.771
Mean ± SD	3.82 ± 1.47	3.94 ± 1.39	
Min–max	1.47–7.0	1.39–7.0	
Financial situation, *n* (%)			0.972
- Very good	7 (17.9)	2 (11.8)	
- Good	18 (46.2)	11 (64.7)	
- Average	12 (30.8)	3 (17.6)	
- Difficult	2 (5.1)	1 (5.9)	
Main source of the mother’s income, *n* (%)			0.504
- Employment contract	17 (43.6)	6 (35.3)	
- Self-employment	3 (7.7)	2 (11.8)	
- Other benefits (social benefits, pension, disability pension, scholarship)	10 (25.6)	2 (11.8)	
- No stable income	5 (12.8)	5 (29.4)	
- Not applicable	4 (10.3)	2 (11.8)	
Main source of the father’s income, *n* (%)			0.807
- Employment contract	25 (64.1)	13 (76.5)	
- Self-employment	7 (17.9)	2 (11.8)	
- Other benefits (social benefits, pension, disability pension, scholarship)	3 (7.7)	1 (5.9)	
- No stable income	3 (7.7)	0 (0)	
- Not applicable	1 (2.6)	1 (5.9)	
Who lives with the child? *n* (%)			1.000
- Only mother	6 (15.4)	3 (17.6)	
- Only father	2 (5.1)	0 (0)	
- Both parents	29 (74.4)	14 (82.4)	
- Other guardian	2 (5.1)	0 (0)	
Who is raising the child? *n* (%)			0.513
- Only mother	6 (15.4)	1 (5.9)	
- Only father	3 (7.7)	0 (0)	
- Both parents	29 (74.4)	16 (94.1)	
- Other guardian	1 (2.6)	0 (0)	
Does the child have siblings? *n* (%)			0.723
- Yes	24 (61.5)	12 (70.6)	
- No	15 (38.5)	5 (29.4)	
Number of siblings, *n* (%)			0.910
- 1	13 (33.3)	5 (29.4)	
- 2	4 (10.3)	5 (29.4)	
- 3	4 (10.3)	1 (5.9)	
- ≥4	3 (7.7)	1 (5.9)	
Number of older siblings, *n* (%)			0.793
- 0	10 (25.6)	2 (11.8)	
- 1	8 (20.5)	8 (47.1)	
- 2	2 (5.1)	1 (5.9)	
- 3	1 (2.6)	0 (0)	
- ≥4	3 (7.7)	1 (5.9)	
Number of younger siblings, *n* (%)			0.935
- 0	11 (28.2)	7 (41.2)	
- 1	11 (28.2)	2 (11.8)	
- 2	0 (0)	3 (17.6)	
- 3	2 (5.1)	0 (0)	

**Table 4:** Data on the childhood of first-degree relatives depending on the presence of feeding disorders.

Parameter	Group A *n* = 39 (100%)	Group B *n* = 17 (100%)	*p*
Mother’s development during childhood, *n* (%) - Normal - Abnormal	36 (92.3) 3 (7.7)	17 (100) 0 (0)	0.546
Father’s development during childhood, *n* (%) - Normal - Abnormal	39 (100) 0 (0)	16 (94.1) 1 (5.9)	0.304
Development of the child’s siblings according to the parent/guardian’s opinion *, *n* (%) - Normal - Abnormal	19 (86.4) 3 (13.6)	12 (100%) 0 (0)	0.537
Was the child’s mother a picky eater during childhood? *n* (%) - Yes - No	17 (43.6) 22 (56.4)	5 (29.4) 12 (70.6)	0.483
Did the child’s mother eat selectively during childhood? *n* (%) - Yes - No	16 (41) 23 (59)	6 (35.3) 11 (64.7)	0.915
Was the child’s father a picky eater during childhood? *n* (%) - Yes - No	8 (20.5) 31 (79.5)	4 (23.5) 13 (76.5)	1.000
Did the child’s father eat selectively during childhood? *n* (%) - Yes - No	12 (30.8) 27 (69.2)	4 (23.5) 13 (76.5)	0.751
Was/Is any of the child’s siblings a picky eater during childhood? ** *n* (%) - Yes - No	6 (25) 18 (75)	5 (41.7) 7 (58.3)	0.751

* For a group of children with feeding disorders *n* = 22, for a group of children without feeding disorders *n* = 12 (this question concerns only children with siblings); ** For a group of children with feeding disorders *n* = 24, for a group of children without feeding disorders *n* = 12.

**Table 5:** Data on comorbidities and specialist assistance depending on the persistence of feeding disorders.

Parameter	Group A *n* = 39 (100%)	Group B *n* = 17 (100%)	*p*
Does the child have any comorbidities? *n* (%) - Yes - No	26 (66.7) 13 (33.3)	8 (47) 9 (53)	0.278
What diseases does the child have, *n* (%) - Cerebral palsy - Epilepsy - Autism spectrum - Other CNS diseases - Anatomical abnormalities in the oral cavity and upper gastrointestinal tract - Food allergies and/or atopic dermatitis - Genetic syndromes - Functional disorders of the gastrointestinal tract - Other	4 (10.3) 6 (15.4) 5 (12.8) 1 (2.6) 3 (7.7) 10 (25.6) 2 (5.1) 4 (10.3) 4 (10.3)	1 (5.9) 3 (17.6) 1 (5.9) 1 (5.9) 3 (17.6) 2 (11.8) 1 (5.9) 0 (0) 1 (5.9)	
Has the child ever received specialist help, *n* (%) - No - Yes	6 (15.4) 33 (84.6)	5 (29.4) 12 (70.6)	0.280
What specialist help did the child receive, *n* (%) - Psychiatric - Psychological - Neurological speech therapy - Dietary - Other	8 (20.5) 16 (41) 24 (61.5) 21 (53.8) 12 (30.8)	3 (17.6) 5 (29.4) 6 (35.3) 7 (41.2) 4 (23.5)	
Has the child’s mother ever sought specialist help, *n* (%) - No - Yes	23 (59) 16 (41)	13 (76.5) 4 (23.5)	0.340
What specialist help did the mother use, *n* (%) - Psychiatric - Psychological - Neurological speech therapy - Dietary	7 (17.9) 10 (25.6) 2 (5.6) 8 (20.5)	2 (11.8) 2 (11.8) 1 (5.9) 1 (5.9)	
Has the child’s father ever sought specialist help, *n* (%) - No - Yes	31 (79.5) 8 (20.5)	15 (88.2) 2 (11.8)	0.706
What specialist help did the father use, *n* (%) - Psychiatric - Psychological - Neurological speech therapy - Dietary - Other	1 (2.6) 4 (10.3) 1 (2.6) 5 (12.8) 1 (2.6)	0 (0) 1 (5.9) 0 (0) 1 (5.9) 0 (0)	
Does the mother think her relationship with her child is satisfactory *, *n* (%) - No - Rather not - Rather yes - Yes	0 (0) 2 (5.6) 11 (30.6) 23 (63.9)	1 (5.9) 0 (0) 1 (5.9) 15 (88.2)	0.422
Does the father think his relationship with his child is satisfactory *, *n* (%) - No - Rather not - Rather yes - Yes	0 (0) 3 (8.3) 13 (36.1) 20 (55.6)	0 (0) 1 (5.9) 4 (23.5) 12 (70.6)	0.525

* *n* = 36 (100%) in the group of children with current feeding disorders.

## Data Availability

The data that support the findings of this study are available from the corresponding author, Martyna Chojnacka, upon reasonable request. The data are not publicly available due to their containing information, which could compromise the privacy of research participants.

## Supplementary Materials

The following supporting information can be downloaded at: https://www.mdpi.com/article/10.3390/nu17071111/s1, Table S1: Data from parents of the studied children depending on the persistence of feeding disorders; Table S2: Data on the past feeding of the studied children depending on the persistence of feeding disorders.

## Abbreviations

The following abbreviations are used in this manuscript:

PFD	Paediatric Feeding Disorder
CM NCU	Collegium Medicum Nicolaus Copernicus University
WHO	World Health Organisation
ICF	International Classification of Functioning, Disability and Health
ARFID	Avoidant/Restrictive Food Intake Disorder
ICD	International Classification of Diseases
BMI	Body Mass Index
SD	Standard Deviation
SID	Sensory Integration Disorder
TV	Television
CP	Cerebral Palsy
