# Supplementary material for: A Preliminary Single-Centre Study on the Risk Factors Associated with Persistent Feeding Disorders in Children
Source: Nutrients. 2025 Mar 22;17(7):1111. doi: 10.3390/nu17071111 (PMC11990817; doi:10.3390/nu17071111)
Supplement: Supplementary file 1 [file nutrients-17-01111-s001.zip › nutrients-3522628-supplementary.pdf]

Table S1. Data from parents of the studied children depending on the persistence of feeding disorders.

| Parameter | Group A<br>N=36 (100%) | Group B<br>N=17 (100%) | p |
|---|---|---|---|
| Mother's data | | | |
| Current age, years<br>mean ± SD<br>min - max | <br>36.64 ± 6.92<br>20.0 – 48.0 | <br>38.29 ± 7.60<br>20.0 – 54.0 | 0.516 |
| Age at birth of the child, years<br>mean ± SD<br>min - max | <br>29.58 ± 5.63<br>19.0 – 47.0 | <br>29.12 ± 5.35<br>19.0 – 40.0 | 0.667 |
| Current weight, kilograms<br>mean ± SD<br>min - max | <br>63.19 ± 8.86<br>45.0 – 80.0 | <br>63.24 ± 12.84<br>48.0 – 95.0 | 0.620 |
| Current height, centimeters<br>mean ± SD<br>min - max | <br>163.67 ± 6.58<br>150.0 – 180.0 | <br>165.35 ± 6.93<br>145.0 – 174.0 | 0.138 |
| BMI<br>mean ± SD<br>min - max | <br>23.64 ± 3.35<br>16.53 – 31.25 | <br>23.26 ± 5.16<br>16.61 – 32.87 | 0.322 |
| Education, n %<br>- Primary<br>- Vocational<br>- Secondary<br>- Post-secondary<br>- Higher | <br>1 (2.8)<br>4 (11.1)<br>10 (27.8)<br>4 (11.1)<br>17 (47.2) | <br>1 (5.9)<br>3 (17.6)<br>4 (23.5)<br>0 (0)<br>9 (52.9) | 0.607 |
| Father's data | | | |
| Current age, years<br>mean ± SD<br>min - max | <br>40.50 ± 8.72<br>21.0 – 63.0 | <br>43.00 ± 9.45<br>23.0 – 60.0 | 0.240 |
| Age at birth of the child, years<br>mean ± SD<br>min - max | <br>33.00 ± 7.09<br>20.0 – 58.0 | <br>33.65 ± 7.07<br>21.0 – 44.0 | 0.573 |
| Current weight, kilograms<br>mean ± SD<br>min - max | <br>84.14 ± 16.98<br>55.0 – 120.0 | <br>83.76 ± 11.93<br>62.0 – 105.0 | 0.927 |
| Current height, centimeters<br>mean ± SD<br>min - max | <br>179.58 ± 7.86<br>165.0 – 200.0 | <br>177.35 ± 8.35<br>160.0 – 191.0 | 0.363 |
| BMI<br>mean ± SD<br>min - max | <br>26.09 ± 5.12<br>16.4 – 39.4 | <br>26.69 ± 3.86<br>19.0 – 33.9 | 0.487 |
| Education, n % | | | 0.761 |

| | | | |
|---|---|---|---|
| - Primary | 1 (2.8) | 2 (11.8) | |
| - Vocational | 9 (25) | 3 (17.6) | |
| - Secondary | 10 (27.8) | 5 (29.4) | |
| - Post-secondary | 1 (2.8) | 0 (0) | |
| - Higher | 15 (41.7) | 7 (41.2) | |

Table S2. Data on the past feeding of the studied children depending on the persistence of feeding disorders.

| Parameter | Group A N=39 (100%) | Group B N=17 (100%) | p |
|---|---|---|---|
| How the baby was fed in the first 3 days of life, n (%) | | | |
| - Only breast milk | 18 (46.2) | 11 (64.7) | 0.372 |
| - Breast milk and modified milk | 11 (28.2) | 4 (23.5) | |
| - Only modified milk | 10 (25.6) | 2 (11.8) | |
| How the baby was fed from 4th day to 6th month of life, n (%) | | | |
| - Only breast milk | 12 (30.8) | 6 (35.3) | 0.758 |
| - Breast milk and infant formula | 16 (41) | 8 (47.1) | |
| - Only infant formula | 11 (28.2) | 3 (17.6) | |
| Type of formula used, n (%) | | | |
| - Regular infant formula | 13 (33.3) | 9 (52.9) | |
| - Milk replacer mixture (for allergy sufferers) | 13 (33.3) | 1 (5.9) | 0.109 |
| - Other infant formula | 1 (2.6) | 1 (5.9) | |
| - Not applicable, baby fed exclusively with mother's milk | 12 (30.8) | 6 (35.3) | |
| Did the mother breastfeed, n (%) | | | |
| - Yes | 30 (76.9) | 14 (82.4) | 0.738 |
| - No | 9 (23.1) | 3 (17.6) | |
| If the mother breastfed, for how long, months | | | |
| mean ± SD | 13.18± 13.08 | 11.57 ± 9.98 | 1.000 |
| min - max | 0.5 – 54.0 | 1.0 – 30.0 | |
| Was breastfeeding painful, n (%) | | | |
| - Yes | 3 (7.7) | 2 (11.8) | 0.647 |
| - No | 27 (69.2) | 12 (70.6) | |
| Have there been any problems with lactation, n (%) | | | |
| - Yes | 10 (25.6) | 6 (35.3) | 0.783 |
| - No | 20 (51.3) | 8 (47.1) | |
| Has the baby choked while feeding, n (%) | | | |
| - Yes | 9 (23.1) | 3 (17.6) | 0.738 |
| - No | 30 (76.9) | 14 (82.4) | |
| Has the child experienced excessive regurgitation, n (%) | | | |
| - Yes | 16 (41) | 5 (29.5) | 0.599 |
| - No | 23 (59) | 12 (70.6) | |

| | | | |
|---|---|---|---|
| Has the child experienced recurrent vomiting, n (%)<br>- Yes<br>- No | <br>8 (20.5)<br>31 (79.5) | <br>4 (23.5)<br>13 (76.5) | 1.000 |
| When the first complementary food was introduced, months<br>mean ± SD<br>min - max | <br><br>7.59 ± 7.34<br>3.0 – 48.0 | <br><br>6.36 ± 1.69<br>5.0 – 12.0 | 0.748 |
| Did the child eat at the end of the first year of life, n (%)*<br>- Foods of all consistencies<br>- Only liquid foods<br>- Liquid and pasty foods | <br>10 (27)<br>7 (18.9)<br>20 (54.1) | <br>9 (64.3)<br>0 (0)<br>5 (35.7) | 0.036 |
| Did the child eat at the end of the first year of life, n (%)*<br>- All foods<br>- Only selected foods<br>- Child did not eat any other food except dairy products | <br>9 (24.3)<br>16 (43.2)<br>12 (32.4) | <br>5 (35.7)<br>8 (57.1)<br>1 (7.1) | 0.184 |
| Did feeding disorders concern milk consumption, n (%)*<br>- Yes<br>- No | <br>16 (43.2)<br>21 (56.8) | <br>6 (42.9)<br>8 (57.1) | 0.770 |
| Did feeding disorders concern other foods, n (%)*<br>- Yes<br>- No | <br>31 (83.8)<br>6 (16.2) | <br>6 (42.9)<br>8 (57.1) | 0.011 |
| When the first problems with food intake occurred, months<br>mean ± SD<br>min - max | <br><br>6.16 ± 7.45<br>1.0 – 36.0 | <br><br>48.21±68.92<br>1.0 – 192.0 | 0.307 |
| Feeding problems occurred, n (%)*<br>- When exclusively breastfeeding<br>- When breastfeeding and formula feeding<br>- When breastfeeding and complementary foods<br>- When feeding with formula and complementary products<br>- When exclusively feeding complementary foods | <br>4 (10.8)<br>3 (8.1)<br>8 (21.6)<br>13 (35.1)<br>9 (24.3) | <br>4 (28.6)<br>2 (14.3)<br>1 (7.1)<br>3 (21.4)<br>4 (28.6) | 0.374 |
| Did the child watch TV/phone while eating, n (%) *<br>- Yes<br>- No | <br>11 (29.7)<br>26 (70.3) | <br>3 (21.4)<br>11 (78.6) | 0.730 |
| Did an infection of the respiratory tract or urinary tract occurred before the onset of feeding disorders, n (%)*<br>- Yes<br>- No | <br><br>4 (10.8)<br>33 (89.2) | <br><br>0 (0)<br>14 (100) | 0.565 |
| How long did it take on average to feed a baby, minutes<br>mean ± SD<br>min - max | <br>38.43±26.46<br>2.0 – 120.0 | <br>30.00 ± 17.65<br>5.0 – 60.0 | 0.388 |
| Did the child like teethers, pacifiers, put objects in the mouth, n (%)*<br>- Yes<br>- No | 24 (64.9)<br>13 (35.1) | 10 (71.4)<br>4 (28.6) | 0.749 |
| Did the child suck a pacifier, n (%)* | | | 0.689 |

| | | | |
|---|---|---|---|
| - Yes<br>- No | 20 (54.1)<br>17 (45.9) | 6 (42.9)<br>8 (57.1) | |
| Has the child ever been fed via tube/PEG, n (%)*<br>- Yes<br>- No | <br>11 (29.7)<br>26 (70.3) | <br>3 (21.4)<br>11 (78.6) | <br>0.730 |

*N=37 in the group of children with current feeding disorders and N=14 in the group of children without current feeding disorders
